# Quantitative HSQC Analyses of Lignin: A Practical Comparison

**DOI:** 10.5936/csbj.201303016

**Published:** 2013-11-10

**Authors:** Marco Sette, Heiko Lange, Claudia Crestini

**Affiliations:** a University of Rome ‘Tor Vergata’, Department of Chemical Sciences and Technologies, Via della Ricerca Scientifica, 00133 Rome, Italy

**Keywords:** natural polymer, structure elucidation, quantitative heterocorrelated NMR, QQ-HSQC, HSQC_0_

## Abstract

Lignin is the second-most abundant polymer after cellulose within the biomass of our planet. Structurally, it displays random oligomeric units without fixed repetition schemes beyond the stage of dimers. Quantitative ^1^H-^13^C HSQC measurements have recently greatly facilitated lignin analyses. In some cases, however, long acquisition times needed for obtaining quantitative HSQCs are not compatible with the chemical integrity of (a potentially functionalised) lignin sample. We thus compared different methods that were developed for more time-efficient quantitative HSQC measurements with respect to their usefulness in lignin analyses: reliable and reproducible results were obtained using both the QQ-HSQC and the HSQC_0_ method.

## Introduction

Wasting’ or ‘leaving unused’ abundant renewable resources is a problem in light of the no longer justifiable use of fossil-based resources with respect to practical, ecological, and socio-economic reasons. Lignin from forest-biomass is one of the naturally abundant resources that are currently seen as waste. Serving both as stabilising and protecting element within the plants, where it is chemically ‘fixed’ in lignin-carbohydrate complexes (LCC), its non-uniform distribution within the plant cell walls sums up to species-specific amounts [1, 2] ranging from 20 ±4% in hardwoods, to 28 ±3% in softwoods and herbaceous angiosperms; monocots generally contain less lignin (15±4%). Obtained mainly as a by-product in the pulp- and paper-industries, its intriguingly divers chemical structure does represent so far a major obstacle with respect to its widespread valorisation beyond the uses as energy source or as substrate for simple bulk chemicals such as vanillin or dimethyl sulfoxide (DMSO) [1, 3]. For solving this dilemma, reliable, easy-to-use, and quick methods are needed that will allow the elucidation of the structural features of a given lignin, since only a sound structural information will guarantee, *i.e*., will allow the design of a (chemical) valorisation strategy that can account in detail for the lignin-typical complexity and heterogeneity.

One of the most reliable methods for investigating structural features of substances, and thus also of lignin, is NMR spectroscopy. Thanks to the advances in the technology as such, it is nowadays possible to gain structural information on lignin at an unprecedented accuracy level, both qualitatively and quantitatively; several articles and scientific monographs on this topic appeared in archival literature in the last years [4–6]. As it has been pointed out before, among others, two-dimensional heterocorrelated NMR spectroscopy advanced the understanding of structural features of lignin, since especially qualitative and quantitative HSQC-analyses shed light on abundances of the different typical interunit bonding units in lignin.

**Figure 1:**
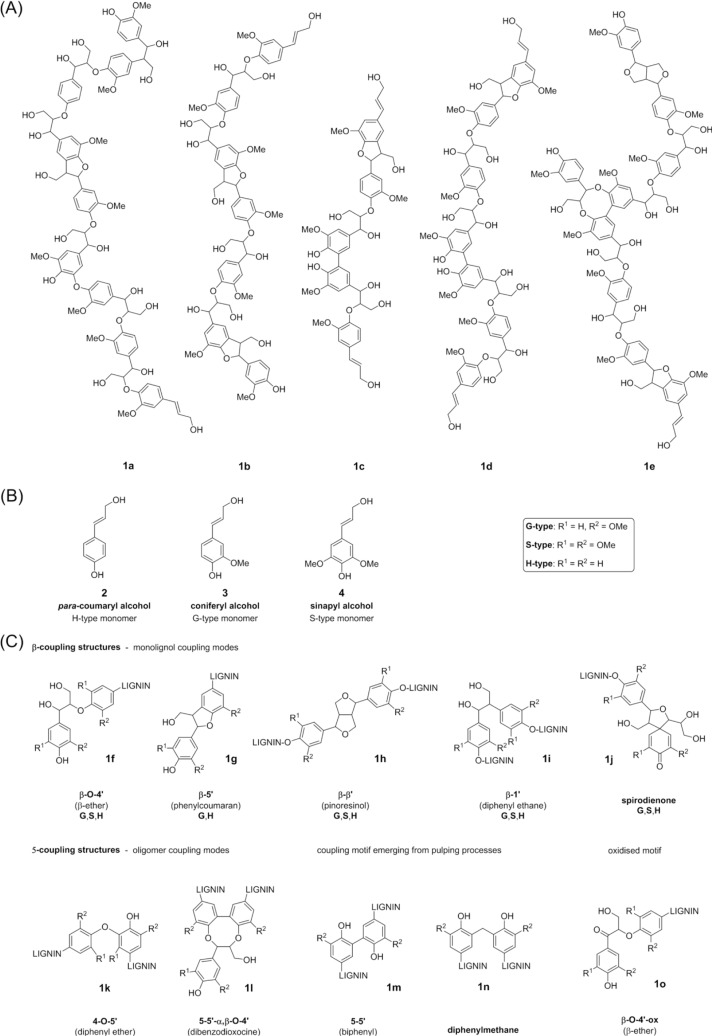
(A) Representative structures of lignin biopolymers, (B) specific lignin types, and (C) main linkage motifs found in lignin and lignin extracts. See main text for details.

Lignin (**1**) is a complex polyphenolic oligomer / polymer, displaying always a plant-specific composition [7, 8]. To the best of the current knowledge, lignin lacks a – in terms of classical polymer-chemistry – defined primary structure, but rather represent random phenyl-propanoid (C9) polyphenols, which are mainly linked by arylglycerol ether bonds between phenolic *para*-coumaryl alcohol (**2**) (H-type), coniferyl alcohol (**3**) (G-type),and sinapyl alcohol (**4**) (S-type) units [9, 10]: lignin of gymnosperms consists almost entirely of G-type lignin (G-lignin); dicotyledonous angiosperms produce a mixture of G- and S-type lignins (GS-lignin). All three types of lignin can be found in variable quantities in monocotyledonous lignin (GSH-lignin). Incomplete or modified monolignols accompany these three main lignin types in woody materials [11]. The diversity in binding types found in lignin stems from its biosynthesis, in which initially formed monolignol radicals re-combine. The resulting dehydrodimer is used by the same enzymes to induce a second round of radical polymerisation, etc: hence lignin formation is not an organised living radical polymerisation, but rather a series of polymerisation termination reactions involving mainly oligomers. The relative abundance of the H/G/S units, in connection with the distribution of different motifs of interunit linkages, which result from the various coupling events, is generally used for chemically and structurally describing a lignin sample. The three monomer units form – as far as structurally possible – up to eight characteristic interunit linkages (Figure 1C). Coupling is generally favoured at the β-position of the monolignol species, resulting in the formation of arylglycerol-β-aryl ethers (β-O-4’ motif, **1f**), phenylcoumarans (β-5’ motif, **1g**), pinoresinols (β-b’ motif, **1h**), diphenylethane dimers (β-1’ motif, **1i**), and spirodienones (SD motif, **1j**). Dilignols and higher oligomers preferentially couple at positions 4 and 5, yielding diaryl ethers (4-O-5’ motif, **1k**), dibenzodioxocines (5,5’-α’β-O-4’ motif, **1l**) and biphenyls (5–5’ motif, **1m**).

Not always mentioned explicitly, but nonetheless important to keep in mind, is the fact that both the mechanical and the chemical means used for isolating the lignin from the biomass source, induce changes to the natural structure of a plant-specific lignin. Besides the industrially important lignins, *Kraft-lignin*, *sulphite-lignin* (*lignosulfonate*), *pyrolisis lignin* and *steam-explosion lignin*, also the *milled wood lignin* (*MWL*), *acidolysis lignin* (*AL*), *cellulolytic enzyme lignin* (*CEL*), *enzymatic mild acidolysis lignin (EMAL) and* *organosolv lignin* show characteristic structural features that are reminiscent of the name-giving isolation procedure; *MWL* commonly serves as the basis for comparison [6, 12]; in fact the oligomer-type lignin structure shown in Figure 1A was derived on the basis of a sample of milled wood lignin [13].

The lack of regular patterns other than the recurring binding motifs mentioned above, is – to the best of our current knowledge and understanding – indeed only the result of the interesting biosynthetic pathway that starts with the generation of the monolignol radicals [14, 15], and proceeds with the recombination of these radicals rather than with a propargation of a classical radical chain reaction. Involving the so-generated dimers, trimers, etc. in the same reaction cycles lead then to the observed oligomeric lignin structures shown in Figure 1A.

As mentioned before, the different structural characteristics of a given lignin sample are best to be elucidated using heteronuclear 2D-NMR techniques, both with respect to qualitative and quantitative analyses. As described in greater detail by others before [4–6], the characterisation of lignin by 2D-NMR techniques has benefitted from both the advancements in NMR technologies as such, and from the advances in delineating and ascribing the NMR signals based on model studies and comparative lignin studies [4,5,6, and cited references]. Several difficulties, however, have to be considered and overcome for achieving a reliable result, especially within quantitative analyses: i) the sample has to be fully dissolvable in the solvent of choice, and stay dissolved during the time of the measurements; ii) standards have to be inert and stable over the time needed for the measurements; iii) signal to noise ratios have to be reasonable within reasonable time-frames needed for the measurement. While the solubility issue can often be resolved by simply acetylating the sample, and /or by working in DMSO-*d6* as solvent, and while optimum external and internal standards have been identified long ago [6], achieving a reasonable S/N-ratio within a reasonable experiment time is still a challenge within the given analyses parameters set by sample stability, solubility etc.

Inspired by similar problems, modified heteronuclear single quantum coherence (2D-HSQC) protocols have been developed by other groups [6, 16–18]. We have recently started to adopt these time- and S/N-optimised methodologies for the examination of MWL lignins, and were able to obtain important structural information using these optimised techniques in connection with other standard techniques used in lignin characterisation [13, 19]. In fact the elucidation of the linearity of the oligomers of MWL lignin was achieved using QQ-HSQC [13, 17]. Although this technique proved very useful, we set out to test another quantitative HSQC-technique, HSQC_0_, which is simpler to implement than the QQ-HSQC method. We report here the first results obtained by the HSQC_0_ method applied in the analyses of an acetylated standard MWL sample that is well studied otherwise. We were especially interested to see whether the different pulse sequences result in generally different quantifications, or whether certain motifs were distinctly affected due to the effects of certain characteristics of the pulse sequences and acquisition methods.

## Experimental Details

### Sample preparation

Milled wood lignin was isolated from Norway spruce wood as reported before [13] by slight modification of the Björkman method [20]. Acetylation was performed in pyridine/acetic anhydride (V/V = 1:1) at 50 °C for 48 h. Ethanol was added, and the volatiles were removed *in vacuo*; the procedure was repeated twice. Then, toluene was added, and the volatiles were quickly removed *in vacuo*, this was repeated twice, as well. Finally, chloroform was added, and the volatiles were removed *in vacuo*; again, this was repeated twice. The solid was then dried overnight *in vacuo* (see Note-1).

### Measurements

All spectra were acquired at 303 K with a Bruker Avance 600 spectrometer equipped with a cryoprobe. The sample consisted of 80 mg of acetylated lignin dissolved in 600 µL of DMSO-*d6*. A matrix consisting of 256 x 2048 points was obtained in eight scans. QQ-HSQC measurements were performed in accordance with the original reference [17] as reported before [13, 19]. In the HSQC_0_-related measurements, the second and third HSQC were obtained as repetitions of the basic HSQC scheme, according to the published procedure [18].

### Data processing

NMR data were processed with MestreNova (Version 8.1.1, Mestrelab Research) by using a 60°-shifted square sine-bell apodisation window; after Fourier transformation and phase correction a baseline correction was applied in both dimensions. The final matrix consisted of 1024 x 1024 points, and cross-peaks were integrated with the same software that allows the typical shape of peaks present in the spectrum to be taken into account. Extrapolations based on the values of the volumes of the peaks of interest of the three consecutive HSQC measurements were performed using MS Excel 2010, to yield the volumes of the different peaks for quantitative analyses.

Raw spectral data and table calculations used for processing are available as Supporting Information or form the authors upon request.

## Results and Discussion

**Figure 2:**
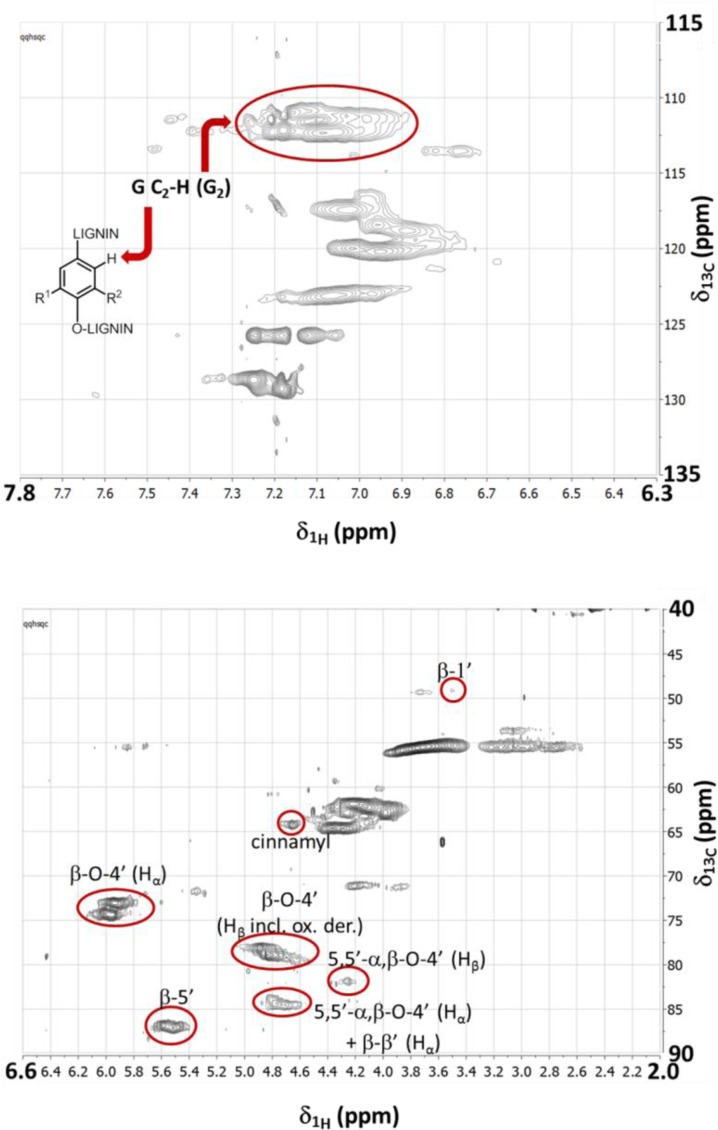
QQ-HSQC contour diagrams obtained for Norway spruce MWL using a 600 MHz NMR spectrometer equipped with a cryoprobe. Signals corresponding to important lignin interunit bonding motifs are indicated (*N.b*: the circles do only indicate the signal positions used for integration, and do not mirror the areas used for quantitative analyses.). Details concerning sample preparation, data acquisition, and data processing are given in the Experimental Details section and in reference [12].

Quantitative CPMG-adjusted HSQC (Q-CAHSQC) measurements [16] were often too long with respect to the integrity of the samples. Quick quantitative HSQC (QQ-HSQC) measurements – as developed by Peterson and Loenig [17] – were thus adopted for measurements of samples of lignin, which we prefer to acetylate before measurement in order to ensure complete solubility of the samples in the NMR solvents (here DMSO-*d6*) at concentrations that should guarantee high signal-to-noise (SN) ratios (see **Note**-1). After careful calibration of pulses, we obtained the best signal-to-noise ratios using a 600 MHz NMR spectrometer equipped with a cryoprobe. We based quantification for softwood lignins on the guaiacyl (G) C_2_-H signals (G_2_) as internal standard (IS), since the C_2_ position of the guaiacyl unit never is substituted, and since it is clearly assignable in the HSQC spectrum. Routinely, though the amount of G_2_ directly reflects the amount of C_9_ units, we validated the quatification using the C2-signals of the C_2_-H signals of both the *para*-hydroxyphenyl (H_2_) and the syringyl (S_2_) units for hardwood and grass lignins. Figure 2 shows exemplary spectra obtained for acetylated Norway spruce milled wood lignin (MWL).

Based on our own experience, as well on standard literature on lignin NMR [4–6, 21], we decided on the integration ranges roughly indicated in Figure 2 (red cycles), and given in the Supporting Information, which led to the absolute intensity values listed in Table 1, where we also list the integration values found by the QQ-HSQC. Very reproducible results were obtained before for this method, with errors of 0.01 – 2 bonds per 100 C_9_ units [19].

**Table 1:** Representative absolute intensities and deduced relative abundances (in% C9) of the internal standard (G C_2_-H (G_2_)) and important interunit bonding motifs in Norway spruce MWL. Samples were acetylated before spectral analysis (see Experimental Details). Abbreviations used in the table: abs. – absolute; rel. – relative; std. – standard; ox. – oxidised.

entry	motif	absolute intensity a		average abs. intensities per group/motif a		rel. abundances [% C9] a	

		QQ-HSQC	HSQC_0_	QQ-HSQC	HSQC_0_	QQ-HSQC	HSQC_0_
1	G2 (G C2-H)	613	11731	613	11731	100	100
2	β-O-4’ (H_α_ of std. motif)	246	4481	246	4481	40.1	38.2
3	β-O-4’ (H_β_ incl. ox. der.)	262	4712	262	4712	42.7	40.2
4	β-O-4’ (C_2_-H of ox. der.)	38	617	38	617	(2.6) b 6.1	(2.0) b 5.3
5	β-5’ (H_α_)	73	1065	73	1065	11.8	9.1
6	β-1’ (H_β_)	10	162	10	162	1.6	1.4
7	5,5’-α,β-O-4’ (H_β_)	16	271	16	271	2.5	2.3
8	cinnamyl alcohol (Hγ)	27	513	14	256	2.2	2.2
9	β-β‘ (H_α_) c	41	834	41	834	6.7	7.1

a Data shown are for one representative set of measurements whose contour diagrams are shown in the pictures. QQ-HSQC and HSQC_0_ measurements were obtained from two distinct acetylation procedures using the very same Norway spruce MWL sample as starting material.

b Values in brackets represent the relative abundance determined by subtracting the relative abundance of the H_α_ of the β-O-4’ standard motif (1f) from that of the H_β_ of the β-O-4’ standard motif that also comprises the H_β_ abundances of the in benzylic position oxidised form of β-O-4’ (motif **1o**).

c Abundances have been determined by subtracting the intensity of 5,5’-α,β-O-4’ (H_β_) from the intensity of the combined signal of [5,5’-α,β-O-4’ (H_α_) + β-β‘ (H_α_)] (HSQC_x_: d_H_ = 4.79, d_C_ = 84.76 ppm; QQ-HSQC: d_H_ = 4.74, d_C_ = 84.50 ppm).

**Figure 3:**
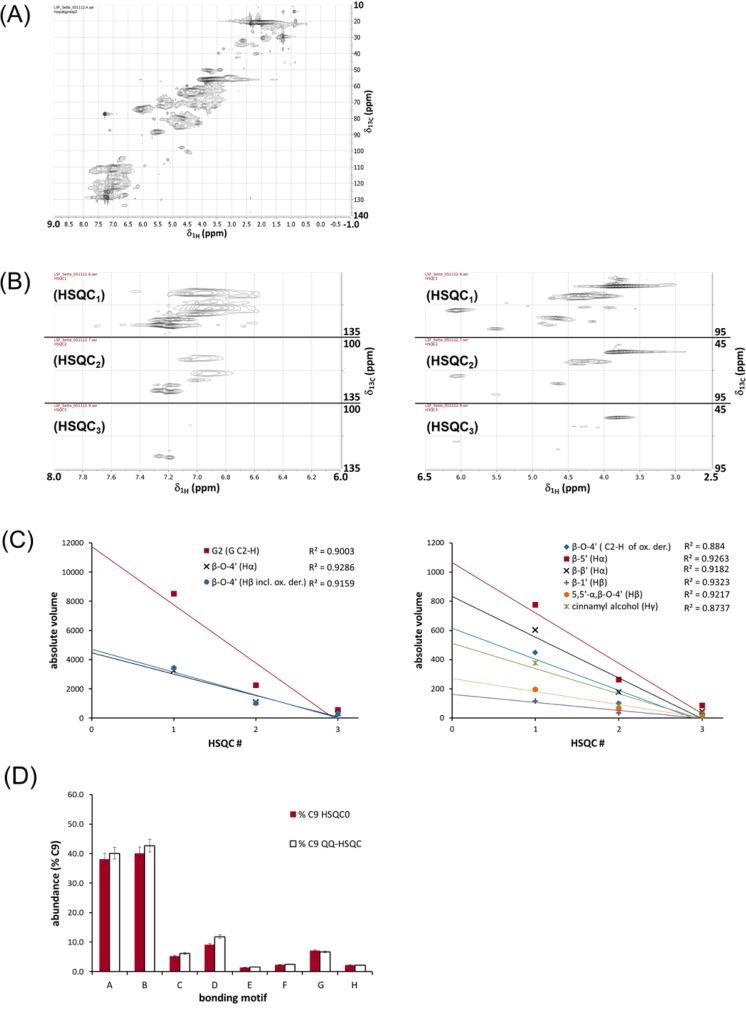
(A) Full range HSQC_1_ obtained for acetylated Norway spruce MWL using a 600 MHz spectrometer equipped with a cryoprobe. (B) Zoom-ins for the aromatic region (*δ*_1H_ 8.0-6.0; *δ*_13C_ 135-100), and the aliphatic region (*δ*_1H_ 6.5-2.5; *δ*_13C_ 95-45) of contour-diagrams obtained for interesting regions for the HSQC_1_, HSQC_2_, and HSQC_3_. (C) Backwards extrapolation for representative signals. (D) Graphical comparison of the intensities of signals corresponding to the main interunit bonding motifs found in the Norway spruce MWL sample (as% C9); legend: A - β-O-4’ (H_α_), B - β-O-4’ (H_β_ including oxidised derivative), C - β-O-4’ ( C_2_-H of oxidised derivative), D - β-5’ (H_α_), E - β-1’ (H_β_), F - 5,5’-α,β-O-4’ (H_β_), G - β-β‘ (H_α_), H - cinnamyl alcohol (H_γ_).

Using these results as a base for further evaluations, we adopted a different sequence for quantitative ^1^H-^13^C HSQC studies that was developed originally by Hu, Westler, and Markley, and that consists of multiple repetitions of a basic HSQC pulse sequence [18]. Using this approach, a so-called HSQC_0_ is obtained by extrapolation of data collected in the aforementioned series of repetitions of a basic HSQC. The experiment starts with a standard HSQC sequence (HSQC_1_, Figure 3A). HSQC_2_ and HSQC_3_ are then obtained by simple double repetition of the basic scheme using incremented repetition times. All the problems associated with quantification in a standard HSQC (*J*-coupling modulation, *T*_2_ relaxation, etc.) produce a linear attenuation of the signals going from HSQC_1_ to HSQC_3_. Following integration of the relevant peaks in the three experiments, an HSQC_0_, *i.e*., a problem-free HSQC, is then obtained by backward extrapolation of the data. Figure 3B shows contour diagrams obtained for three consecutive HSQC spectra obtained for the same acetylated Norway spruce MWL lignin analysed before with the QQ-HSQC pulse sequence (Figure 2). Exemplary extrapolation curves obtained by integration of the signals the internal standard (G C_2_-H (G_2_)) and important interunit bonding motifs in Norway spruce MWL for HSQC_1_, HSQC_2_, and HSQC_3_ are shown in Figure 3B; Figure 3C shows the graphical analyses towards the determination of intensities in the theoretical HSQC_0_. In general, the quality of the linear regressions used for the backward extrapolation is acceptable (R^2^ ≥ 0.9); only two extrapolations of signals of lower intensities, namely for the oxidised form of the interunit bonding motif β-O-4’ (**1o**), and for the cinnamyl endgroups (groups **2-4** (Figure 1) linked *via* an ether bond to a lignin backbone) result in poorer coefficients of determination (R^2^ = 0.88, 0.87, respectively). Whereas the difficulties in the determination of the cinnamyl endgroups might result from the fact that quantification is based on a CH_A_H_B_-group rather than a simple CH-group, problems in the quantification of the oxidised β-O-4’ unit are likely to result from overlaps. The quality of the latter can be estimated by comparing the relative abundance with the difference between the relative abundances found for the joint signal of the H_β_ of both the β-O-4’ motif (**1f**) and its oxidised analogue **1o**, and the signal for the H_α_ of the β-O-4’ motif (**1f**): for both methods, this deduction of the relative abundance of the oxidised form **1o** of the β-O-4’ motif (**1f**) results in only half the amount. Based on the quality of the spectral data, however, one of the two ways for determining the amount of oxidised β-O-4’ units is more reliable; in the present case, the high purity of the MWL sample used renders the substraction-method more viable.

The HSQC_0_-intensities are listed in Table 1 for comparison with the previously obtained data. Figure 3D represents an additional graphical comparison of the results obtained for the most important interunit bonding motifs in lignin.

The direct comparison of the data obtained using the two different methods for obtaining quantitative HSQC data reveals the same relations between important interunit bonding motifs. Furthermore, also the results of the quantifications against the internal standard (G C_2_-H (G_2_)) are showing essentially the same results, considering the experimental errors in the measurements for all of the motifs investigated except the β-5’ (H_α_) motif. This motif, however, is known to be quantified only with difficulties. Given the difficult nature of the lignin as such, our results indicate that both quantitative HSQC methods deliver very similar, and thus comparable results, and that both can be used in lignin analyses. Comparing the two methods, with respect to the time that is actually needed for obtaining the spectra, and with respect to the plain key figures of the acquisitions, etc., it has to be clear that the easier to be implemented HSQC_0_ is a series of three consecutive HSQC measurements, in which each experiment has the same relaxation delay, the same number of scans and the same number of experiments as the QQ-HSQC: in our case, 16 scans were accumulated using a relaxation delay of 12 seconds over a matrix consisting of 2048 x 256 points. Thus, the total acquisition time of HSQC_0_ experiment is three times the experimental time of the QQ-HSQC.

Having obtained good results using the HSQC_0_ method on a 600MHz spectrometer equipped with a cryoprobe, we tested whether comparable results can be obtained running the HSQC_0_ program on a more common 400 MHz spectrometer without cryoprobe. The results we obtained using this machine were unfortunately not matching the above reported results, the main difficulties arising from very unsatisfying S/N ratios obtained within the HSQC_2_ and HSQC_3_ measurements (data not reported). We then increased the number of scans in connection using a non-uniform sampling scheme [22]. Unfortunately, also this method, at the end, did not work using a 400 MHz NMR spectrometer equipped with a standard broadband (BBO) probe. Further studies are currently under way using a 400 MHz NMR spectrometer equipped with a cryoprobe, in order to elucidate the importance of the field strength for these types of measurements.

Concluding, we found that both, QQ-HSQC and HSQC_0_ as standard methods for obtaining quantitative HSQC data, can be successfully exploited for lignin analyses, delivering generally comparable data, with QQ-HSQC being the faster method, while the HSQC_0_ method is easier to implement. Further trials aiming at reducing the time needed for obtaining a quantitative HSQC spectrum for a standard lignin sample, by using non-uniform sampling techniques in connection with the HSQC_0_ method, did not result in viable results.

## Supplementary Material

Quantitative HSQC Analyses of Lignin: A Practical Comparison

## Note-1

We perform the acetylation as outlined in the Experimental Details as smoothly as possible, using rotary vane pumps for achieving a good vacuum that ensures a minimum of time for, and low temperatures during the evaporation of solvents within the work-up sequence, in order to avoid undesired structural changes within the lignin sample.

## References

[1] Aresta M, Dibenedetto A, Dumeignil F editors (2012) Biorefinery: From biomass to chemicals and fuels Berlin/Boston: de Gruyter 445 p.

[2] Sannigrahi, P, Pu, Y, Ragauskas, A (2010). Cellulosic biorefineries - unleashing lignin opportunities. Curr Opin Environ Sustain 2: 383–393

[3] Glasser WG, Northey RA, Schultz TP editors (1999) Lignin: Historical, biological and materials perspectives. ACS Symposium Series 742 Washington, DC: American Chemical Society 559 p.

[4] Landucci LE (1989) Chapter 3 - Search for Lignin Condensation Reactions with Modern NMR Techniques In: Heminigway RW, Conner AH, Branham SJ editors. Adhesives from renewable resources ACS Symposium Series 385. Washington, DC: American Chemical Society pp. 27–42

[5] Ralph J, Landucci LL (2011) NMR of Lignins In: Heitner C, Dimmel DR, Schmidt JH editors. Lignin and Lignans: Advances in Chemistry. Boca Raton: CRC Press, Taylor & Francis Group pp. 137–244

[6] Wen, JL, Sun, SL, Xue, BL, Sun, RC (2013) Recent Advances in Characterization of Lignin Polymer by Solution-State Nuclear Magnetic Resonance (NMR) Methodology. Materials 6: 359–391 10.3390/ma6010359 PMC5452107 28809313

[7] Marton J (1966) Lignin structure and reactions. Advances in Chemistry, Volume 59 Washington, DC: American Chemical Society

[8] Holtman, H, Chang, M, Jameel, H, Kadla, JF (2003) Elucidation of lignin structure through degradative methods: Comparison of modified DFRC and thioacidolysis. J Agric Food Chem 51: 3535–3540 12769520 10.1021/jf0340411

[9] Early review: Adler, E (1957) Structural elements of lignin. Ind Eng Chem 49: 1377–1383

[10] Lewis NG, Sarkanen S editors (1998) Lignin and Lignan Biosynthesis. ACS Symposium Series 697 Washington, DC: American Chemical Society 436 p.

[11] Vanholme, R, Demedts, B, Morreel, K, Ralph, J, Boerjan, W (2010) Lignin Biosynthesis and Structure. Plant Physiol 153: 895–905 20472751 10.1104/pp.110.155119 PMC2899938

[12] Lange, H, Decina, S, Crestini, C (2013) Oxidative upgrade of lignin – Recent routes reviewed. Eur Polym J 49: 1151–1173

[13] Crestini, C, Melone, F, Sette, M, Saladino, R (2011) Milled wood lignin: A linear oligomer. Biomacromolecules 12: 3928–3935 21928799 10.1021/bm200948r

[14] Whetten, R, Sederoff, R (1995) Lignin biosynthesis. Plant Cell 7: 1001–1013 12242395 10.1105/tpc.7.7.1001 PMC160901

[15] Boerjan, W, Ralph, J, Baucher, M (2003) Lignin biosynthesis. Annu Rev Plant Biol 54: 519–546 14503002 10.1146/annurev.arplant.54.031902.134938

[16] Koskela, H, Kilpeläinen, I, Heikkinen, S (2005) Some aspects of quantitative 2D NMR. J Magn Reson 174: 237–244 15862240 10.1016/j.jmr.2005.02.002

[17] Peterson, DJ, Loening, NM (2007) QQ-HSQC: a quick, quantitative heteronuclear correlation experiment for NMR spectroscopy. Magn Reson Chem 45: 937–941 17924357 10.1002/mrc.2073

[18] Hu, K, Westler, WM, Markley, JL (2011) Simultaneous Quantification and Identification of Individual Chemicals in Metabolite Mixtures by Two-Dimensional Extrapolated Time-Zero ^1^H-^13^C HSQC (HSQC_0_). J Am Chem Soc 133: 1662–1665 21247157 10.1021/ja1095304 PMC3037033

[19] Sette, M, Wechselberger, R, Crestini, C (2011) Elucidation of lignin structure by quantitative 2D NMR. Chem Eur J 17: 9529–9535 21721058 10.1002/chem.201003045

[20] Björkman, A. (1956) Studies on finely divided wood. Part 1. Extraction of lignin with neutral solvents. Svensk Papperstidn 59: 477–85

[21] Rencoret, J, Gutiérrez, A, Nieto, L, Jiménez-Barbero, J, Faulds, CB (2011) Lignin Composition and Structure in Young versus Adult Eucalyptus globulus Plants. Plant Physiology 155: 667–682 21098672 10.1104/pp.110.167254 PMC3032458

[22] Maciejewski, MW, Mobli, M, Schuyler, AD, Stern, AS, Hoch, JC (2012) Data Sampling in Multidimensional NMR: Fundamentals and Strategies. Top Curr Chem 316: 49–77 21773916 10.1007/128_2011_185 PMC6078388

